# Inferring Economic Impacts from a Program’s Physical Outcomes: An Application to Forest Protection in Thailand

**DOI:** 10.1007/s10640-021-00644-z

**Published:** 2022-02-06

**Authors:** Wumeng He, Orapan Nabangchang, Krista Erdman, Alex C. A. Vanko, Prapti Poudel, Chandra Giri, Jeffrey R. Vincent

**Affiliations:** 1 Economics and Management School, Wuhan University, Wuhan, Hubei China; 2 School of Economics, Sukhothai Thammathirat Open University, Pakkret Nonthaburi, Thailand; 3 South Dakota Department of Agriculture, Pierre, SD USA; 4 Wildlands Network, Durham, NC USA; 5 College of Natural Resources, North Carolina State University, Raleigh, NC USA; 6 U.S. Environmental Protection Agency, Durham, NC USA; 7 Nicholas School of the Environment, Duke University, Durham, NC 27708 USA

**Keywords:** Forest, Impact evaluation, Mangrove, Propensity score matching, Protected area, Random utility model, Roy model, Thailand, Treatment effect

## Abstract

Economists typically estimate the average treatment effect on the treated (ATT) when evaluating government programs. The economic interpretation of the ATT can be ambiguous when program outcomes are measured in purely physical terms, as they often are in evaluations of environmental programs (e.g., avoided deforestation). This paper presents an approach for inferring economic impacts from physical outcomes when the ATT is estimated using propensity-score matching. For the case of forest protection, we show that a protection program’s ex post economic impact, as perceived by the government agency responsible for protection decisions, can be proxied by a weighted ATT, with the weights derived from the propensity of being treated (i.e., protected). We apply this new metric to mangrove protection in Thailand during 1987–2000. We find that the government’s protection program avoided the loss of 12.8% of the economic value associated with the protected mangrove area. This estimate is about a quarter smaller than the conventional ATT for avoided deforestation, 17.3 percentage points. The difference between the two measures indicates that the program tended to be less effective at reducing deforestation in locations where the government perceived the net benefits of protection as being greater, which is the opposite of the relationship that would characterize a maximally effective program.

## Introduction

Economists routinely use treatment effects models to evaluate government programs’ causal impacts on observed outcomes (Imbens and Wooldridge 2009; Athey and Imbens 2017). Identifying causal relationships requires addressing various potential sources of bias, including non-random selection, confounding, and spillover effects. Economists have developed a variety of quasi-experimental impact evaluation methods to address these biases, including matching, instrumental variables, regression discontinuity, and difference-in-differences. These methods implicitly assume that the economic outcomes of interest are observable. In some settings, however, economists only observe outcomes measured in physical terms, which might lack well-defined economic interpretations. Although impact evaluations using physical outcomes might be feasible, their results do not necessarily serve as useful proxies for programs’ economic impacts. This paper presents a method for inferring the economic impact of a program when outcomes are observed in physical terms.

We present this method in the context of forest conservation and demonstrate its empirical application by evaluating a forest zoning system in Thailand that aimed at protecting mangrove forests. Mangroves are coastal ecosystems located in the tropics and subtropics that provide a wide range of benefits to local and global populations. In a series of papers, Mäler and colleagues listed these benefits as including the production of shellfish, firewood, timber, and game; feeding and nursery areas for commercially important fish; shoreline and storm protection; nesting, resting, and feeding sites for birds; and carbon sequestration (Mäler et al. 1996, 2008, 2009; Arrow et al. 2000). A large literature confirms the economic importance of these benefits (Himes-Cornell et al. 2018), including in Thailand (Barbier 2007).^1^

Private land owners, however, often have incentives to convert forests, including mangroves, to more profitable land uses. Since the free market likely fails to supply the socially optimal amount of forest, governments often intervene by implementing forest conservation programs, including protected areas, payments for ecosystem services, and community-based forest management. Most impact evaluations of these programs use forest cover as the outcome variable and aim to measure a program’s causal effect on avoided deforestation (Blackman 2013; Lan and Yin 2017). An impact evaluation of this type defines a program’s average treatment effect on the treated (ATT) as the difference between the realized forest cover change in treated areas—i.e., sites where the program was implemented—and the counterfactual forest cover change that would have occurred in the same areas had they not been treated.

The ATT for avoided deforestation is a good proxy for a forest conservation program’s economic impact only if the benefits and costs of conservation are homogeneous (Vincent 2016). Environmental economics theory has recognized that environmental values can instead vary spatially and that government programs to protect the environment must account for this variation since at least Mäler’s (1974) seminal treatise. There is abundant evidence that both the benefits (e.g., carbon sequestration, Asner et al. 2010; biodiversity habitat, Gibson et al. 2011; Le Saout et al. 2013; watershed services, Pattanayak and Kramer 2001; Brauman et al. 2007) and the costs (Naidoo et al. 2006; Polasky 2008) of conservation are highly heterogeneous. Mäler et al. (2008; 2009) emphasize that the economic benefits of maintaining mangroves are “very case sensitive” and can vary greatly by location due to differences in ecological, economic, and institutional conditions. A program that increases forest cover by, for instance, 20% thus does not necessarily increase forest value by 20% too. Depending on the distribution of benefits and costs across the sites included in a program, the ATT for avoided deforestation can either overestimate or underestimate the ATT for a program’s economic impact.

We develop an indirect way to estimate the ATT for a forest conservation program’s economic impact by building on work by Heckman (2010), who emphasized the structural framework embedded in treatment effects models. We employ a discrete-choice Roy (1951) model to describe the process of selection into treatment. One can view this model as a random utility model (RUM; McFadden 1973; Manski 1977) and parametrically estimate the effects of forest attributes on behavior in response to a conservation program. A byproduct of this procedure is the estimated propensity score. We show that under certain conditions, a transformation of the propensity score has a utility interpretation, where utility is as perceived by the government agency that decides which forests to include in the program. We use this transformation as a weight to construct a utility-weighted ATT. This new metric can be viewed as a generalized form of the ATT for avoided deforestation, and it offers a better measure of the ATT for a forest conservation program’s economic impact, at least as perceived by the government.

Our method connects the impact evaluation literature to the non-market valuation literature. Propensity score matching is a common impact evaluation technique, and random utility models are widely applied in valuing non-market goods (Freeman et al. 2014). We show how to combine these two approaches and exploit their respective advantages. In doing so, we contribute to filling a gap in the environmental policy analysis literature, in which valuation and evaluation are disconnected (Ferraro et al. 2012).

Our empirical analysis focuses on mangrove protection in Thailand. In 1987, recognizing both the high value of Thailand’s mangroves and their rapid loss (Charuppat and Charuppat 1997), the Thai government introduced a zoning system for managing the nation’s mangroves (Havanond 2008). This system assigned mangroves to different categories that varied in terms of restrictions on forest use, including conversion to non-forest land uses. We evaluate the zoning system using both the ATT for avoided deforestation and the utility-weighted ATT that we develop, and we compare the results. The utility-weighted ATT indicates that the system avoided the loss of 12.8% of the economic value associated with the protected mangrove area. This impact is about a quarter smaller than the conventional ATT for avoided deforestation, 17.3 percentage points. The difference between the two measures indicates that the zoning system tended to be less effective at reducing deforestation in locations where the government perceived the net benefits of protection as being greater. This relationship is the opposite of what one would expect for a maximally effective program.

The rest of the paper is organized as follows. Section 2 presents the theoretical foundations and econometric aspects of our method. Section 3 applies the method to the mangrove zoning system in Thailand. The last section discusses the interpretation of the utility-weighted ATT and its limitations as an evaluation metric.

## Deriving and Estimating a Utility-Weighted ATT

Impact evaluations are program-specific, and the empirical strategy one should use to identify program impacts depends on program features and the data structure. Although our proposed method can in principle be applied to non-environmental programs, to explain its derivation and estimation we refer to the evaluation of a hypothetical forest protection program, with propensity score matching as the evaluation strategy. We begin with an intuitive explanation of the method before presenting details on its derivation and estimation.

### The Intuition for a Utility-Weighted ATT

Avoided deforestation (*Q*), a binary variable that equals 1 if deforestation does not occur (i.e., forest is retained) and 0 otherwise, is the most widely used outcome variable in the impact evaluation literature on forest protection programs. To estimate the ATT for avoided deforestation ($\tau _{ATT^Q}$), economists first observe the realized forest outcomes for a sample of protected (“treated”) sites, then use a technique such as matching to construct for each treated site a counterfactual (i.e., the hypothetical forest outcome had the site not been treated), and finally compute an unweighted average of the differences between the realized outcomes and the counterfactuals across all treated sites. In this approach, all treated sites have the same weight; they are not weighted by the utility gains from protecting them. Hence, the total amount of effective protection–protection that actually results in avoided deforestation–affects the ATT, but which sites are protected effectively does not. Table 1 presents a simple numerical example of the evaluation of a program that protects three sites. We consider three hypothetical cases in which protection is effective at only one of the sites. When all sites have the same weight, $\tau _{ATT^Q}$ equals one-third in each case, regardless of which site is protected effectively.

Even if the sites have the same area, the utility gains from protecting them could vary. We assume that effective protection generates the least gain at Site A and the greatest gain at Site C. We can incorporate these heterogeneous values into the evaluation by constructing a utility-weighted ATT ($\tau _{ATT^W}$), which is lowest when only Site A is protected effectively (Case 1) and highest when only Site C is protected effectively (Case 3). Compared to the conventional evaluation metric $\tau _{ATT^Q}$, this utility-weighted metric $\tau _{ATT^W}$ conveys more information on the economic performance of protection. As Table 1 illustrates, $\tau _{ATT^W}$ can be larger than, smaller than, or equal to $\tau _{ATT^Q}$, depending on which sites are protected effectively and how utility gains vary across them.

Site-level protection values are often not directly observable. We develop a new approach to overcome this data limitation within an impact evaluation framework by inferring the values in an indirect way. If the government protection agency (the “regulator”) makes rational protection decisions, then we can model their decision process by a RUM and estimate each site’s propensity score for being protected. A higher propensity score implies a higher expected utility gain as viewed by the regulator. Below, we formalize this approach and show how to derive site-level utility weights based on the propensity score and use them to estimate $\tau _{ATT^W}$.

### Behaviors of the Land User and the Regulator

We use the Roy model to explain forest-related behavior first for a private land user and then for a regulator. Assume there is a collection of forest sites, with individual sites distinguished by *i*, and for simplicity a single land user who decides whether to deforest each site. $Q_i=0$ if deforestation occurs and $Q_i=1$ otherwise. Unless necessary for clarity, we suppress the site index *i* henceforth to simplify notation. *X* denotes a set of site characteristics known to both the land user and the regulator. In the absence of regulation, the land user’s private utilities with and without deforestation are given respectively by $Y^L_0$ and $Y^L_1$,

1 $$\begin{aligned} Y^L_0&= \mu ^L_0(X) + U^L_0 \end{aligned}$$

2 $$\begin{aligned} Y^L_1&= \mu ^L_1(X) + U^L_1 \end{aligned}$$

$U_0^L$ and $U_1^L$ denote utility effects known only to the land user; both have expected values of zero. These effects could include the land user’s idiosyncratic knowledge of market conditions for products extracted from mangroves or supplied by non-mangrove land uses, or non-market uses of mangroves unknown to the regulator.

In the presence of regulation, a rational deforestation decision implies

3 $$\begin{aligned} Q = \mathbf {1}(Y^L_1 > Y^L_0) \end{aligned}$$

where

4 $$\begin{aligned} Q = D Q_{1} + (1 - D) Q_{0} \end{aligned}$$

with *D* being the treatment status ($D=1$ if the site is protected, $D=0$ otherwise) and *Q*_1_ and *Q*_0_ being deforestation outcomes with and without protection, respectively. The regulator (and econometricians) can observe *Q* but not $Y^L_0 - Y^L_1$, the private utility gain if deforestation occurs. As written, Eq. (3) includes no penalty for noncompliance with the protection regulation. Absent such penalty, the land user has no incentive to modify their behavior compared to the unregulated market setting.

Turning to the behavior of the regular, we initially follow the mangrove management example in Arrow et al. (2000) by assuming the regulator aims to increase social welfare, which mirrors Mäler’s (1974) assumption of an environmental management agency with knowledge of society’s preferences. Recognizing the existence of a social benefit from retaining forest that the land user ignores, the regulator has an incentive to intervene and correct the market failure by protecting sites. The regulator is rational and protects a site if and only if the social benefit from retaining forest exceeds the expected private gain from deforestation (i.e., the expected opportunity cost of protection),

5 $$\begin{aligned} D = \mathbf {1} \Big (\mu ^S(X) + U^S > k^R\big (\mu ^L_0(X) - \mu ^L_1(X) \big )\Big ) \end{aligned}$$

The social benefit is on the left side of the inequality and has two components: $\mu ^S(X)$, which is determined by observed site characteristics; and $U^S$, which is the regulator’s private knowledge about the social benefit and has an expected value of zero. The right side shows the expected private gain from deforestation, $\mu ^L_0(X) - \mu ^L_1(X)$, multiplied by a scalar $k^R$ that converts the expected private gain to the same units as the social benefit; note that it does not account for the unobserved (by the regulator and econometricians) component of private utility. We can rewrite this expression as

6 $$\begin{aligned} \begin{aligned} D&= \mathbf {1} \Big ( \mu ^S(X) - k^R\big (\mu ^L_1(X) - \mu ^L_0(X) \big )> - U^S \Big )\\&\equiv \mathbf {1} \Big ( \mu ^R(X) > U^R \Big ) \end{aligned} \end{aligned}$$

where the second line uses more compact notation to relabel terms on the first line. We refer to this expression as the selection equation.

To induce compliance, regulation imposes a penalty, such as a fine or lost profits, that the land user must pay if they are caught deforesting ($Q=0$) a protected site ($D=1$). We assume the land user can form an unbiased estimate of these costs, $C>0$, by multiplying their subjective probability of being caught by a prediction of the penalty. Because *C* is estimated by the land user, it is not observed by the regulator (or econometricians). Its addition changes Eq. (3) to

7 $$\begin{aligned} Q = \mathbf {1} \Big ( Y^L_{1} > Y^L_{0} - D C \Big ) \end{aligned}$$

which we can rewrite as

8 $$\begin{aligned} Q = D \times \mathbf {1} \Big ( \mu ^L(X)> U^L - C \Big ) + (1 - D) \times \mathbf {1} \Big ( \mu ^L(X) > U^L \Big ) \end{aligned}$$

We group *U* and *C* on the right hand side of the inequalities to highlight that they pertain to the land user’s private information.

### Evaluating the Program’s Net Economic Impact

We now shift the perspective from the regulator’s ex ante protection decisions to ex post evaluation of the protection program. If the outcome variable is forest status *Q*, then the individual treatment effect can easily be defined based on Eq. (8),

9 $$\begin{aligned} \tau _i = Q_{1i} - Q_{0i} = \mathbf {1} \Big ( \mu ^L(X_i)> U^L_i - C \Big ) - \mathbf {1} \Big ( \mu ^L(X_i) > U^L_i \Big ) \end{aligned}$$

To interpret $\tau _i$, we categorize all treated sites into three groups:

**S1**: sites that will not be deforested regardless of being treated or not, i.e., $Q_{1i} = Q_{0i} = 1$;
**S2**: sites that will be deforested regardless of being treated or not, i.e., $Q_{1i} = Q_{0i} = 0$;
**S3**: sites that will not be deforested only if they are treated, i.e., $Q_{1i} = 1$ and $Q_{0i} = 0$.^2^

From Eq. (5), the regulator views the net economic gain from protecting site *i* as the difference between the site’s social benefit, which is lost if the site is deforested, and the expected private gain from deforestation. Denote this difference by $\Delta Y_i$. For sites in group **S1**, the land user voluntarily chooses not to deforest even in the absence of regulation, and so protection causes neither a gain in social benefits nor a loss in land user utility:

10 $$\begin{aligned} \Delta Y_i (i \in \mathbf {S1}) = 0 \end{aligned}$$

For sites in group **S2**, the land user always deforests. Protection still does not cause a gain in social benefits, but now it causes a loss in land user utility due to the additional cost of deforestation, *C*, in the presence of regulation:

11 $$\begin{aligned} \Delta Y_i (i \in \mathbf {S2}) = - k^R C \end{aligned}$$

As noted earlier, the regulator does not observe this cost, a point to which we will return. Finally, for sites in group **S3**, protection causes both a gain in social benefits and a loss in land user utility:

12 $$\begin{aligned} \Delta Y_i (i \in \mathbf {S3}) = \mu ^S(X_i) + U_i^S - k^R \Big ( \mu ^L(X_i) - U_i^L \Big ) \end{aligned}$$

The program’s overall economic treatment effect is determined by summing the impacts across all sites in all three groups:

13 $$\begin{aligned} \tau _{\Delta Y} = \sum _{i \in \mathbf {S2}, D_i=1} (-k^R C) + \sum _{i \in \mathbf {S3}, D_i=1} \Big ( \mu ^S(X_i) + U_i^S - k^R \mu ^L(X_i) \Big ) \end{aligned}$$

We have used here the assumption that the expected value of $U^L_i$ is zero.

$\tau _{\Delta Y}$ can be interpreted as the social welfare gain if the regulator is benign, but it lacks this welfare interpretation if the regulator is not benign. In the latter case, it measures the net economic gain according to the regulator’s preferences, which could differ from society’s preferences. We will offer some insights into this discrepancy for mangrove protection in Thailand by comparing the government’s stated criteria for protection to the factors that our estimated selection equation indicates actually influenced protection decisions.

### Empirical Identification of the Utility-Weighted ATT

Empirical identification of $\tau _{\Delta Y}$ faces four challenges. The first is that *C* is private information known to the land user but not econometricians. The version of $\tau _{\Delta Y}$ that can be estimated will consequently omit the first sum in Eq. (13),

14 $$\begin{aligned} \tau _{\Delta Y} = \sum _{i \in \mathbf {S3}, D_i=1} \Big ( \mu ^S(X_i) + U_i^S - k^R \mu ^L(X_i) \Big ) \end{aligned}$$

This expression represents an upper bound on the true value of $\tau _{\Delta Y}$. The difference between the estimated and true values of $\tau _{\Delta Y}$ is smaller if $k^R C$ is smaller, which occurs if the regulator places lower weight on land user utility, enforcement is weaker, or the penalty is lower. It is also smaller if *C* is a fine whose revenue is returned to the public (a transfer payment) or funds another government program that generates an economic gain.

The second challenge is that $\tau _{\Delta Y}$ has a range of $\mathbb {R}^{+}$. This range prevents an ATT based on it from being compared to the ATT for avoided deforestation. The latter is the expectation of Eq. (9) conditional on treatment,

15 $$\begin{aligned} \tau _{ATT^Q} = \mathbf {E} [Q_1 - Q_0 | D = 1] \end{aligned}$$

which is just the share of **S3** among the treated sites,

16 $$\begin{aligned} \tau _{ATT^Q} = \frac{\sum _{i \in \mathbf {S3}, D_i=1} 1}{\sum _{D_i = 1} 1} \end{aligned}$$

$\tau _{ATT^Q}$ thus ranges from zero to one. We rescale $\tau _{\Delta Y}$ to the same range by applying the following expression,

17 $$\begin{aligned} \tau _{ATT^W} = \frac{\sum _{i \in \mathbf {S3}, D_i=1} \Big ( \mu ^S(X_i) + U_i^S - k^R \mu ^L(X_i) \Big )}{\sum _{D_i=1} \Big ( \mu ^S(X_i) + U_i^S - k^R \mu ^L(X_i) \Big )} \end{aligned}$$

The denominator represents the maximal utility gain that can possibly be achieved, while the numerator represents the actual gain. The “W” in $\tau _{ATT^W}$ indicates that this economic ATT weights treated sites differently from each other, unlike the physical $\tau _{ATT^Q}$ (see Table 1).

The third challenge is that $\tau _{\Delta Y}$ and its rescaled version $\tau _{ATT^W}$ include direct measures of utility, which econometricians do not observe. Empirical identification of $\tau _{ATT^W}$ requires identifying the conditional expectation of the expression in parentheses in it, which we label $W_i$:

18 $$\begin{aligned} W_i \equiv \mathbb {E} [\mu ^S(X_i) + U^S_i - k^R \mu ^L(X_i) | X_i = x, D_i = 1] \end{aligned}$$

Despite its inclusion of direct utilities, $W_i$ can be identified as follows. In the selection equation (Eq. (6)), we assume that $\mu ^R(X)$ can be modeled as a linear function of *X*, $\theta X$, and that $U^R$ follows the standard logistic distribution. These assumptions enable us to identify $\theta$ by estimating the selection equation as a logistic regression model. With $\theta$ identified, we can identify $W_i$ by applying the expression $W_i = \mathbb {E} [\theta X_i + U^S_i | \theta X_i + U^S_i > 0]$, which we show in Appendix 1 is equivalent to

19 $$\begin{aligned} W_i = \frac{e^{\theta X_i}}{1 + e^{\theta X_i}} \ln (1 + e^{\theta X_i}) \end{aligned}$$

$W_i$ is monotonically increasing in $\theta X_i$, which implies that, compared to $\tau _{ATT^Q}$, $\tau _{ATT^W}$ gives more weight to treated sites associated with higher expected utility gains. It follows that $W_i$ is also monotonically increasing in the propensity score from the logistic model,

20 $$\begin{aligned} p(X_i) = \frac{\exp {(\theta X_i)}}{1 + \exp {(\theta X_i)}} \end{aligned}$$

as substituting this expression into Eq. (19) yields

21 $$\begin{aligned} W_i = p(X_i) \ln \Big ( \frac{1}{1 - p(X_i)} \Big ) \end{aligned}$$

The final challenge is the familiar one with impact evaluations: the econometrician observes treated sites only in their treated state, not their counterfactual untreated state, and faces an associated selection problem because both treatment status and the treatment effects are functions of *X*. We address this challenge by using the propensity score to select a matched set of untreated observations as controls for the treated observations. Propensity score matching eliminates selection bias if: (i) selection is only on observables, $(Q_1, Q_0) \perp D|X$ (the Conditional Independence Assumption, CIA; Rosenbaum and Rubin 1983); and (ii) the characteristics of treated and untreated sites overlap sufficiently so that the treated sites and their matched controls are statistically indistinguishable, $\mathbb {P}(D=1|X=x) \in (0,1)$ for all *x* in the support of *X* (the common support assumption; Ho et al. 2007). The CIA is satisfied if $U^S \perp U^L$, which requires including all variables that influence selection and outcomes in the estimated selection equation, and full support can be checked empirically by examining the balance of the treatment and control groups.

Empirical identification of $\tau _{ATT^W}$ thus involves first estimating the selection equation (Eq. (6)), then using Eq. (21) to estimate $W_i$, and finally using propensity score matching to estimate

22 $$\begin{aligned} \hat{\tau }_{ATT^W} = \sum _i \frac{D_i \hat{W}_i}{\sum _i D_i \hat{W}_i} (Q_i - Q_j) \end{aligned}$$

where $Q_j$ is the matched counterpart of $Q_i$. Compared to the estimated version of the ATT for avoided deforestation (Eq. (16)), which is

23 $$\begin{aligned} \hat{\tau }_{ATT^Q} = \sum _i \frac{D_i}{\sum _i D_i} (Q_i - Q_j) \end{aligned}$$

the only difference is the inclusion of the estimated utility weights.

We close with three associated empirical points. First, Appendix 2 shows how the expressions for $\hat{\tau }_{ATT^Q}$ and $\hat{\tau }_{ATT^W}$ given by Eqs. (22) and (23) can be extended to cases in which *Q* is a continuous, not binary, variable. Second, Appendix 3 shows how to estimate the standard error of $\hat{\tau }_{ATT^W}$. Third, Eqs (22) and (23) imply that the magnitude of $\hat{\tau }_{ATT^W}$ relative to $\hat{\tau }_{ATT^Q}$ depends on the correlation between the estimated individual treatment effects ($Q_i - Q_j$) and the estimated propensity scores ($\hat{p}(X_i)$). $\hat{\tau }_{ATT^W}$ is larger (smaller) than $\hat{\tau }_{ATT^Q}$ if the correlation is positive (negative), and the two measures are identical if the correlation is zero. This relationship enables one to predict the potential range of $\hat{\tau }_{ATT^W}$ for a particular empirical case, by using the estimated individual treatment effects and varying the correlation from −1 to +1. One can therefore not only estimate $\hat{\tau }_{ATT^W}$ but also determine if it is closer to the minimal or maximal values it potentially could have had.

## Empirical Application: Mangrove Protection in Thailand

### Policy Context

In the 1970s and 1980s, Thailand experienced a boom in shrimp farming, which entailed the complete or near-complete conversion of affected mangrove areas into shrimp ponds. Mean annual mangrove deforestation rose sharply, from 45 km^2^/yr during 1961–1979 to 130 km^2^/yr during 1979–1986 (Charuppat and Charuppat 1997). By 1986, nearly half (47%) of the country’s original mangrove area had been converted to other land uses, with shrimp farms accounting for a third of the cumulative historical loss (Charuppat and Charuppat 1997). In response to this rapid and large loss, the country’s highest governmental authority, the Prime Minister’s Cabinet, issued a resolution on December 15, 1987 that established a new national mangrove management system. This system assigned the country’s mangroves to three management zones: (1) Protection Zone, which prohibited deforestation, defined as the conversion of mangroves to other land uses, and all resource extraction; (2) Economic Zone A, which prohibited deforestation but allowed sustainable harvesting of wood products by local communities and commercial companies; and (3) Economic Zone B, which allowed both deforestation and extractive uses. The government implemented this system for 13 years. A Cabinet resolution on August 22, 2000 marked its end, by prohibiting the renewal of harvesting contracts in Economic Zone A and any additional deforestation or resource extraction in Economic Zone B. In 2002, the government shifted responsibility for mangrove management from the Royal Forestry Department (RFD) to a new Department of Marine and Coastal Resources (DMCR).

We evaluate Thailand’s zoning system using both the conventional ATT for avoided deforestation and the utility-weighted ATT, with propensity score matching as the impact evaluation method and satellite data on deforestation during 1987–2000. Five features of the zoning system make it attractive for illustrating the application of the utility-weighted ATT. First, consistent with the description of the “regulator” in Sect. 2.2, the Cabinet can be viewed as a single agent that made a discrete protection intervention. This situation differs from more complex ones with protected areas established by multiple bodies (e.g., national, subnational) at multiple points in time. Second, the system had well-defined start and end dates. The delineation of the pre-treatment and treatment periods and the appropriate endpoint for measuring the system’s outcomes are both clear, which is necessary for an impact evaluation.

Third, the system included a zone—the Protection Zone—that prohibited deforestation and thus represents a suitable “treatment” for an impact evaluation with avoided deforestation as the physical outcome measure. Although Economic Zone A prohibited deforestation too, it is not suitable for evaluation because it allowed sustainable wood harvesting. Distinguishing harvesting from deforestation is difficult in satellite imagery before a forest regenerates from harvesting. Including Economic Zone A in the treatment group would risk misclassification of tree-cover loss and biased estimates of treatment effects.

Fourth, using the Protection Zone as the treatment requires information on the spatial locations of mangroves assigned to it, and such information exists because the 1987 Cabinet resolution approved and endorsed a specific map prepared by the RFD. This map delineated the boundaries of all three zones in the entire national area estimated as originally having been mangrove. Cabinet resolutions from 1982 and 1984 had called for the development of a zoning system (Mangroves for the Future 2011). The RFD’s mapping effort stemmed from these resolutions and was completed before the 1987 resolution. The zone assignments remained unchanged during 1987–2000, which implies that using a single selection model—i.e., the first-stage logistic regression model in propensity score matching—to analyze the selection process is appropriate.

Finally, a suitable set of control locations exists because the RFD’s 1987 map omitted some mangroves. We interviewed two senior officers in the DMCR who were members of the RFD team that prepared the map,^3^ and they reported that these omissions were inadvertent. A likely reason is the relatively low resolution of the satellite data that the RFD used for mapping mangroves in the 1980s (1:250,000; Charuppat and Charuppat 1997). Lying entirely outside the zoning system, these omitted mangroves represent a purer control group than do mangroves in Economic Zone B, where deforestation was allowed but required the RFD’s permission.

To our knowledge, the zoning system has never been evaluated using a causal-inference method. In fact, some literature mischaracterizes it as consisting of two zones, not three (Aksornkoae 2004). Case studies of small numbers of villages dominate the literature on mangrove management in Thailand (e.g., two villages, Sudtongkong and Webb 2008; four villages, Barbier 2008 and Kongkeaw et al. 2019). They lack the representativeness necessary for evaluating the national performance of the zoning system, but they offer reasons to doubt the system’s effectiveness at reducing deforestation. They highlight a gap between its progressive aspects, which on paper signaled a policy shift away from conversion toward conservation and gave local communities a greater role in managing mangroves than did corresponding policies for terrestrial forests (Kongkeaw et al. 2019), and its implementation, which by 1996 included communities in the management of barely 5% of the total zoned area (Charuppat and Charuppat 1997). They document disputes between communities and the government over ownership of mangroves, which created de facto open access (Sudtongkong and Webb 2008; Barbier 2008), and a lack of response by the government when communities reported illegal conversion or harvesting (Sudtongkong and Webb 2008). Consistent with this last point, the DMCR officers we interviewed reported that monitoring and enforcement of the system were weak due to inadequate staffing and budget and that penalties, if assessed at all, were typically low.

Despite these deficiencies, the system might not have been completely ineffective: (Charuppat and Charuppat 1997) reported that mean annual mangrove deforestation fell by nearly 80% during the decade after the 1987 Cabinet resolution compared to the decade before. There is thus merit in determining the system’s effectiveness during its entire implementation period, in both biophysical and economically more relevant terms.

### Data and Covariate Construction

**Fig. 1:**
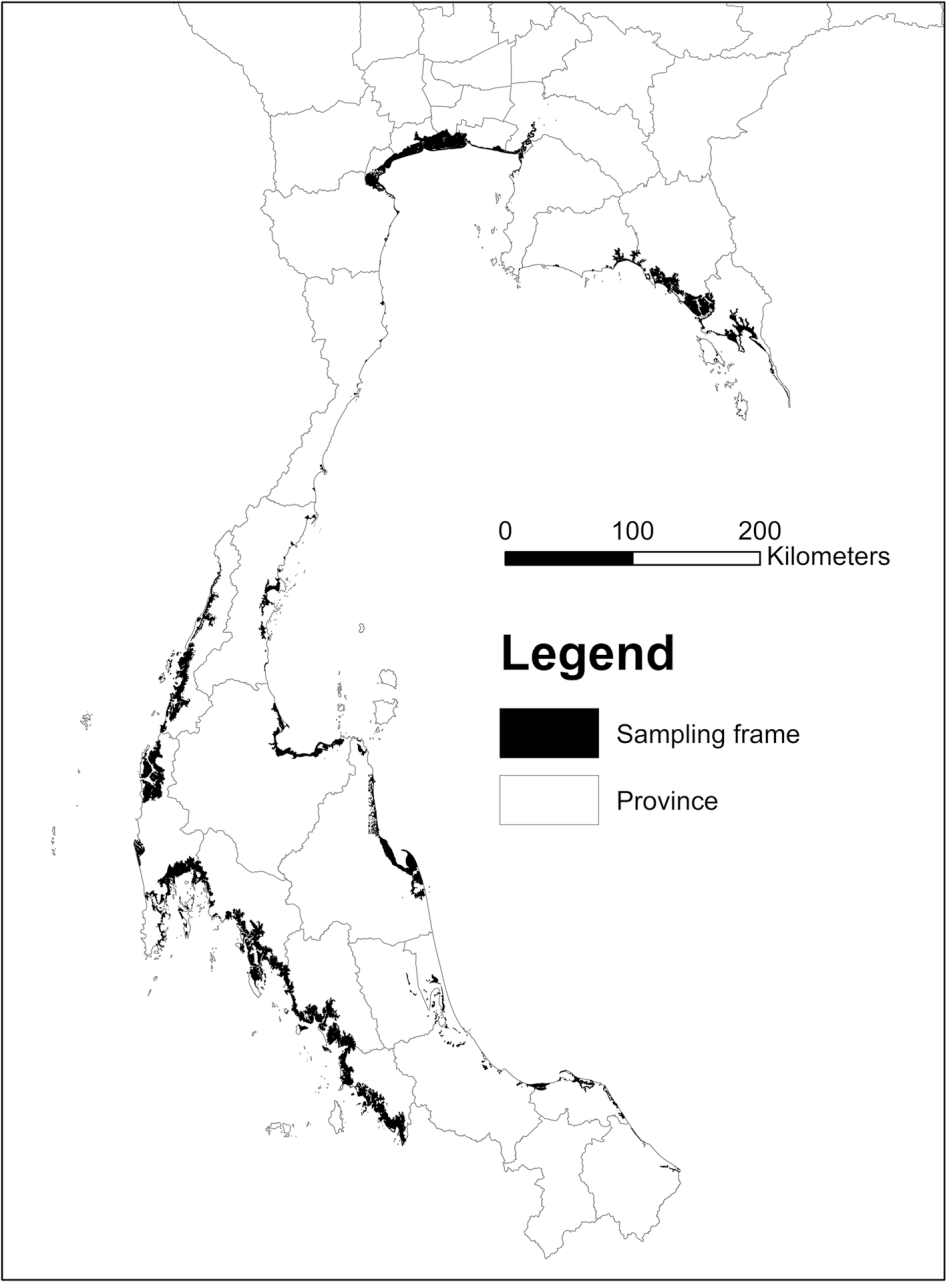
Map of mangrove sampling frame. *Note*: Sampling frame (i.e., areas with mangrove cover in either 1987 or 2000) is shown in black. Gray boundaries demarcate Thai provinces

We highlight key features of our data here, with full details on sources and processing reported in Appendix 4. We obtained a digital map showing mangrove presence/absence for Thailand in 2000 from Giri et al. (2005, 2011). We created a corresponding 1987 map using the same type of satellite data (Landsat), same techniques, and same resolution (30 meter) used to create the 2000 map. Using these maps, we drew a random sample of 50,000 points from locations where mangroves were present in at least one of the two years (see Fig. 1). We set the minimum distance between any two sampled points at 50 meters to prevent drawing multiple points from a single pixel. Given our focus on avoided deforestation, we dropped all points where mangroves were not present in 1987.^4^

**Fig. 2:**
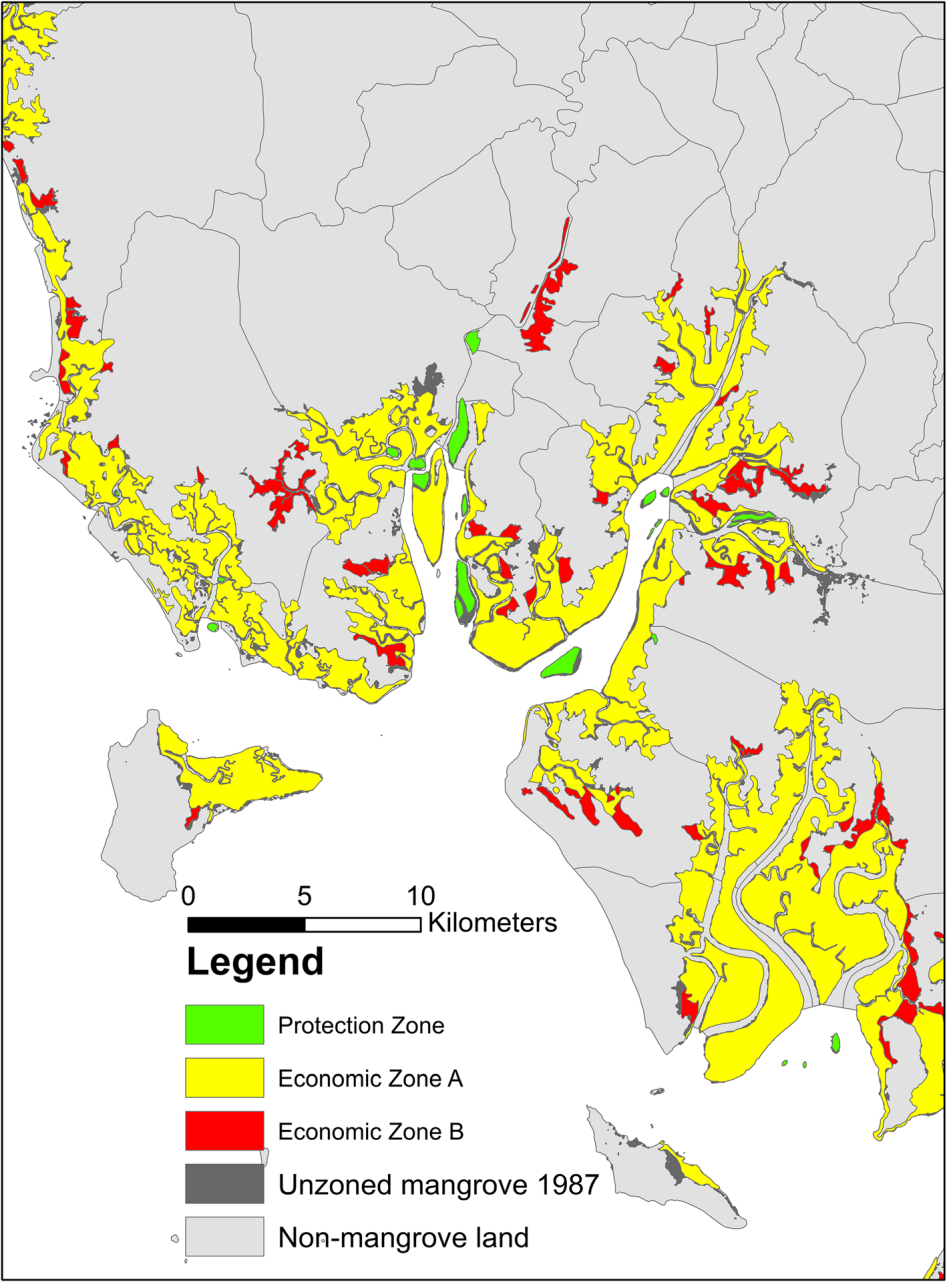
Local illustration of the mangrove zoning system (portion of Krabi Province on Thailand’s Andaman Sea coast)

We classified the treatment status of the sampled points using a shapefile from the DMCR that showed the zone boundaries from RFD’s 1987 map. The map delineated the zones at a fine scale (Fig. 2). The Protection Zone included 524 distinct polygons (noncontinguous areas), which were on average very small (mean and median areas = 0.28 km^2^ and 0.026 km^2^, respectively). It accounted for only 4% of the total zoned area. Overlaying the 1987 mangrove cover map on the zone map revealed that the total area of the unzoned mangrove area omitted from the zone map was equivalent to 26% of the total zoned area. Overlays also revealed that the cumulative 1987–2000 deforestation rate was much lower in the Protection Zone (17%) than in the unzoned area (48%). Although the difference in these rates suggests that assignment to the Protection Zone might have cut deforestation by nearly two thirds, determining the actual impact requires controlling for selection bias through estimation of the ATT for avoided deforestation.

We retained points in the Protection Zone as the treatment group and points in the unzoned area as a pool of potential controls. The 1987 Cabinet resolution stated specific criteria for assigning mangroves to the Protection Zone. Table 2 lists these criteria, which reflect various environmental public goods associated with mangroves and thus imply a social welfare foundation for the criteria. We based our choice of covariates for the selection model on these criteria, the interview with the DMCR officers, and the matching literature on protected areas (PAs). The officers reported that the RFD relied most heavily on criteria 1.6 (existing PAs) and 1.10 (proximity to river banks and coastlines) in preparing the zone map. This reliance is understandable for two reasons. The first is access to spatial information: according to the DMCR officers, the RFD used official PA maps included in the Royal Gazette notices that established the PAs, and it used high-quality military topographical maps to measure distances. Second, these two criteria overlap with several others, with 1.6 subsuming 1.4, 1.5, 1.8, and 1.9 and 1.3 providing the coastal-protecton rationale for 1.10. We dropped points that were in PAs established before the Cabinet resolution (criterion 1.6), as they did not represent incremental treatment by the zoning system. These points accounted for 49% of the treatment group. We incorporated criterion 1.10 by constructing two distance variables, one for distance to the nearest river and the other for distance to the coast.^5^

Regarding the remaining criteria, we incorporated 1.7 by constructing two dummy variables, which distinguished points with higher and lower average wind speeds, respectively, from points with intermediate average wind speeds, and a dummy for points located outside the monsoon climate zone.^6^ We incorporated 1.1 and 1.2 by constructing an index of the number of mangrove-resident animal species whose ranges encompassed each point.

The 1987 Cabinet resolution did not state comparable criteria for Economic Zones A and B, but its authorization of these two zones implies that assignment of mangroves to the Protection Zone was also influenced by the opportunity cost of protection, with protection being less likely in locations where resource extraction and conversion to non-mangrove land uses were more valuable to local communities or industries. Consideration of the opportunity cost of protection is consistent with a net social welfare concept that accounts for more than just the public good benefits of protection reflected in the stated criteria in Table 2. It is also consistent with the PA matching literature, which provides ample evidence that various socioeconomic variables can affect either protection or avoided deforestation and thus are important to include in a selection model. We included a dummy variable to identify points on the Gulf of Thailand coast, where the shrimp farm industry was more heavily concentrated (Charuppat and Charuppat 1997). Although Thailand’s original mangrove area was nearly evenly split between the two coasts (Charuppat and Charuppat 1997), our 1987 mangrove map revealed that the Gulf coast’s share had fallen to barely a third (36%) by that year. This concentration implies a higher opportunity cost of protection on the Gulf coast and thus a lower likelihood of protection there. We also included distance variables of the types commonly used to measure proximity to features that affect the opportunity cost of protection (Pfaff et al. 2009, 2015): distances to the province capital and district (*amphoe*) seat, distance to the nearest road, and a pair of dummy variables that distinguished points nearer to and farther from the forest edge from points at an intermediate distance from the edge. Following Miteva et al. (2015), we also included population density for the subdistrict (*tambon*) where a point was located and the fraction of subdistrict area that was in mangrove in 1987,^7^ both of which can likewise be expected to affect the opportunity cost of protection. Most of these socioeconomic variables can be given a political interpretation in addition to a purely economic one (i.e., opportunity cost). For example, the Gulf dummy could proxy for the political influence of the shrimp farm industry. For this reason, statistical significance of these variables in the estimated selection model could reflect political influence over zoning decisions, which could cause the decisions to deviate from welfare-maximizing ones.

We did not include two variables commonly used in the PA evaluation literature, slope and elevation, because mangroves occur primarily in intertidal zones where variation in these factors is negligible.^8^ The total number of covariates in our selection model, 14, is typical for the literature (Miteva et al. 2015, 13 covariates; Ahmadia et al. 2015), 15 covariates).

Matching requires the common support assumption: covariates must have sufficient overlap between the treatment group and the untreated group. For each covariate, we identified untreated points with values more than 5% beyond the range of treated points and dropped them from the sample. Our final data set included 7,790 points, 671 of them treated. The pool of potential controls was thus large relative to the number of treatment points, which is advantageous for matching. Table 3 lists the covariates and their means and standard deviations for the treated and untreated points. Substantial differences can be observed for many of the variables, which highlights the need for matching to construct a balanced sample before estimating the treatment effects.

### Evaluation Based on ATT for Avoided Deforestation

We used matching with replacement, so that each untreated point could be used as the best match (“nearest neighbor”) for multiple treated points. We set the caliper—the maximum permissible difference between the propensity score for a treated point and its matched control—at 0.01. Application of the caliper reduced the number of treated points by a small amount, from 671 to 653 if we matched each treated point with a single control point and 646 if we matched it with two.

We evaluated covariate balance using the two most common diagnostics in the matching literature: the standardized mean difference between the treatment and control groups, and the ratio of variances for the two groups (Austin 2009). Formal statistical tests of these diagnostics are not valid in propensity score matching (Imai et al. 2008), so we instead applied guidelines recommended in the literature. We required the absolute value of the standardized mean difference to be below 0.1 (Normand et al. 2001; Austin 2009) and the variance ratio to be within the 2.5th and 97.5th percentiles of an *F*-distribution with degrees of freedom implied by the number of matched treatment-control pairs (Austin 2009). These percentiles were 0.86 and 1.17 in our sample. With the number of matched controls per treated point set initially at one, one covariate, *Wind speed: high*, was unbalanced according to the standardized mean difference (0.148) and barely balanced according to the variance ratio (1.165) (see Appendix 5). In response, we increased the number of matched controls to two, which eliminated the imbalance (Table 4). We used two matches in all subsequent analyses.

The literature on PA evaluation emphasizes the need to address spillover effects, which can bias estimates of treatment effects (Robalino et al. 2017; Herrera et al. 2019). There are two types of spillovers, leakage and blockage (Fuller et al. 2019). In the case of leakage, PA establishment displaces deforestation that would have occurred inside a PA to an unprotected location where deforestation would not have occurred in the absence of protection. Conversely, in the case of blockage, PA establishment increases the cost of deforestation in unprotected locations and reduces the amount of deforestation that would have otherwise occurred in those locations. Failure to control for leakage and blockage can result in overestimation and underestimation, respectively, of PAs’ effectiveness in reducing deforestation.

When investigating local spillovers, the definition of “local” depends on the geographical scale of the PA in question. Because mangroves naturally occur in narrow coastal strips, Thailand’s zoning system delineated the polygons for the Protection Zone at a much finer scale (see Fig. 2) than, say, the scale of PAs commonly analyzed in countries such as Brazil (Herrera et al. 2019) and Costa Rica (Robalino et al. 2017). As noted earlier, the mean and median areas of Protection Zone polygons were substantially smaller than 1 $\mathrm {km}^2$. We therefore expect spillover effects to occur primarily within a few kilometers of the corresponding polygons. To investigate the magnitude of spillovers, we first estimated $\tau _{ATT^Q}$, the treatment effect for avoided deforestation, using all 646 matched pairs as a baseline. Then, we progressively excluded untreated points that were within a 1 km, 2 km, 3 km, 4 km, or 5 km buffer of a Protection Zone polygon and, at each step, reestimated the selection model, formed a new set of controls in view of the reduced pool of potential controls, and reestimated $\tau _{ATT^Q}$. Table 5 displays the results. Imposing the buffers only slightly affected the estimates of $\tau _{ATT^Q}$, and the confidence intervals overlap for all the estimates. We therefore conclude that spillover effects were negligible and that the no-buffer case yields the preferred estimate of $\tau _{ATT^Q}$.

In addition to the lack of evidence of spillovers, we prefer the no-buffer case because it includes the largest number of treated points, which falls to only 437 for the 5 km buffer. It also has the best balance: the number of covariates that violate the balance conditions rises from zero for this case to 3, 2, 2, 6, and 7, respectively, for the 1 km, 2 km, 3 km, 4 km, and 5 km buffers. This deterioration in match quality is due to the shrinking pool of potential controls.

From Table 5, the estimated $\tau _{ATT^Q}$ for the no-buffer case has a highly significant (*p* < 0.01) value of 0.173. This estimate indicates that 17.3% of the Protection Zone prevented deforestation that would have occurred in the absence of protection. It is barely half of the 31-percentage point difference between the observed 1987–2000 deforestation rates for the Protection Zone and the unzoned area reported earlier, which underscores the need to control for selection bias when evaluating forest protection programs: due to selection bias, the observed difference in deforestation rates exaggerates the Protection Zone’s impact on avoided deforestation. Nevertheless, the positive and significant value of the estimated $\tau _{ATT^Q}$ confirms that creation of the Protection Zone reduced mangrove deforestation during 1987-2000.^9^

### Evaluation Based on Utility-Weighted ATT

To implement our new evaluation approach based on $\tau _{ATT^W}$, we estimated individual propensity scores for all treated points using the same logistic regression that served as the first stage of our matching estimator. We used the no-buffer sample given its superior balance and the lack of evidence of spillover effects. Table 6 presents the regression results. As discussed in Sect. 3.2, the first six covariates pertain to assignment criteria for the Protection Zone stated in the 1987 Cabinet resolution. Most of them are not significant at even *p* < 0.1. *Wind speed: low* is significant at that level and has the expected negative sign. *Animal species* is highly significant (*p* < 0.01) but is unexpectedly negatively signed: mangroves were less likely to be assigned to the Protection Zone if they had more species. In sum, we found little evidence that the stated criteria influenced treatment.

We found more evidence that socioeconomic covariates mattered. As expected, the Gulf dummy had a negative effect (*p* < 0.1). The probability of being treated was higher for interior mangroves and lower for mangroves near the forest edge (*p* < 0.01 for both) and lower for locations with higher population density (*p* < 0.05), which mirrors results in previous matching studies on PAs (e.g., Joppa and Pfaff 2009; Miteva et al. 2015). We cannot determine whether the significance of these variables reflects rational consideration of the opportunity costs of protection, which would be consistent with the general design of the zoning system, or instead reflects political influences that caused treatment to deviate from social welfare maximization. The lack of evidence that the stated criteria for the Protection Zone mattered inclines us to believe that treatment decisions were not purely apolitical, however.

Using these results, we followed the approach explained in Sect. 2.3 to construct the utility weights for all treated observations in the sample. As explained in Sect. 2.4, the magnitudes of $\tau _{ATT^W}$ and $\tau _{ATT^Q}$ differ when predicted individual treatment effects and propensity scores have a non-zero correlation. When predicted individual treatment effects are positively (negatively) correlated with propensity scores, $\tau _{ATT^W}$ is larger (smaller) than $\tau _{ATT^Q}$. Because $\tau _{ATT^Q}$ does not depend on this correlation, we can readily determine the potential range of $\tau _{ATT^W}$ for the Protection Zone. In effect, we can simulate a more complex, and more realistic, case than the simple example given in Table 1. Holding $\tau _{ATT^Q}$ constant at its estimated value and using the utility weights we constructed, we found that $\tau _{ATT^W}$ attains a maximum value of 0.749 if the two series are perfectly positively correlated and a minimum value of -0.514 if they are perfectly negatively correlated. If we rule out protection causing deforestation of mangroves that would not be deforested in the absence of protection (see Sect. 2.3), then the minimum value becomes zero. $\tau _{ATT^W}$ therefore has a potential range from zero to nearly four times $\tau _{ATT^Q}$ (0.749 vs. 0.173). This wide range, straddling $\tau _{ATT^Q}$, demonstrates that the ATT for avoided deforestation can be a highly misleading proxy for the economic impact of a forest conservation program.

**Fig. 3:**
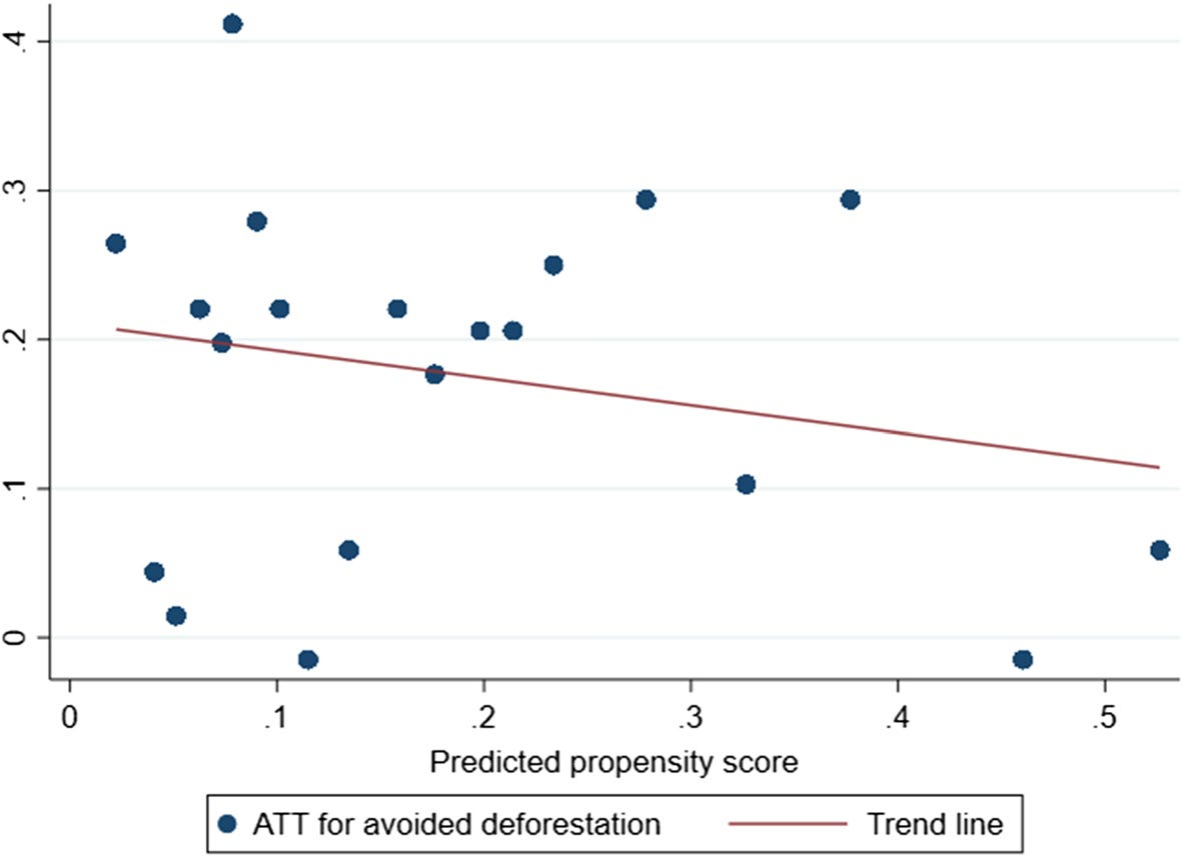
Distribution of the average treatment effect for avoided deforestation ($\tau _{ATT^Q}$) by quantiles of the predicted propensity score (20 quantiles)

Our estimate of the utility-weighted ATT turned out to be near the lower end of the potential range, 0.128. This estimate is approximately a quarter (26%) less than the estimated ATT for avoided deforestation and is highly significant (*p* < 0.01). Figure 3, which plots quantiles of the predicted individual treatment effects for points in the treatment group against the corresponding predicted propensity scores, illustrates that the estimate of $\tau _{ATT^W}$ is smaller than the estimate of $\tau _{ATT^Q}$ for the reason just given: i.e., the two series are negatively correlated.^10^ The two measures have different interpretations: while the estimate of $\tau _{ATT^Q}$ indicates that approximately 17% of the Protection Zone prevented deforestation that would have occurred in the absence of protection, the estimate of $\tau _{ATT^W}$ indicates that the Protection Zone protected approximately 13% of the economic value, as perceived by the government, that would have been lost if all mangroves in the Protection Zone had been deforested. They are not alternative measures of the same quantity.

The difference in magnitude between the two measures suggests that interpreting $\tau _{ATT^Q}$ as a proxy for the Protection Zone’s economic impact would overestimate the impact by roughly a third (35%). The difference is not significant, however (*p* = 0.16). The lack of significance is due primarily to the relatively large standard errors of $\tau _{ATT^Q}$ and $\tau _{ATT^W}$, 0.024 and 0.041, respectively, which in turn has two causes. One cause that is common to both estimates is the relatively small number of treated points in the sample. Tripling the number, from 646 to 1,938, would cause the test statistic to become significant at *p* < 0.04. The other cause, which pertains to $\tau _{ATT^W}$, is that unequal weights can yield less precise estimates than equal weights (see Appendix 3). The weights included in the estimate of $\tau _{ATT^W}$ are unequal, having a coefficient of variation of 1.46.

## Conclusion

Propensity score matching is one of the most widely applied methods for identifying and estimating treatment effects models, especially in the forest conservation literature. In its standard application, the first stage of a propensity score matching study serves only as a statistical step for predicting an index, the propensity score, that is subsequently used to match treated observations to controls. We have argued that when the outcome variable is a binary variable that characterizes behavioral responses by agents to a government program, an economic interpretation is embedded in the first-stage analysis as it reflects the regulator’s preferences in assigning treatment. We exploit this economic interpretation to develop a method, based on utility weights derived from the predicted propensity scores for treated observations, that indirectly evaluates the economic impact of a program whose observed outcomes are measured in physical, not economic, terms. In so doing, we connect the impact evaluation literature to the non-market valuation literature.

If the regulator’s preferences align with society’s, then the utility-weighted ATT generated by our method has a social welfare interpretation. Of course, such alignment does not necessarily occur in practice. For the case of mangrove protection in Thailand during 1987–2000 that we use to demonstrate the method’s empirical application, we show how one can gain insight into the degree of alignment by first reviewing the stated criteria for treatment and then comparing those criteria to estimation results for the selection model. In the Thailand case, the stated criteria for protecting mangroves plausibly aligned with social welfare, as they accounted for both a range of environmental public goods supplied by mangroves and the opportunity costs of protection. On the other hand, selection into treatment was generally not significantly influenced by covariates representing environmental public goods; instead, it was mainly influenced by covariates that represented opportunity costs and possibly political influences. We would thus argue that the utility-weighted ATT in this case measures the economic gain from treatment valued according to government preferences that did not align perfectly with social preferences.

The utility-weighted ATT expands the toolkit that applied economists have for evaluating the impacts of government programs, forest conservation programs in particular. Although we have focused on the evaluation of programs whose observed physical outcomes are binary, we have shown how it can be extended to continuous outcomes (Appendix 2). Nonetheless, it has limitations. First, in the absence of information on enforcement actions against noncompliance with a program, it is best interpreted as an upper bound on economic impacts. This bias is probably small in the context of mangrove protection in Thailand during 1987-2000, as enforcement was reportedly weak and penalties low. Second, derivation of the utility weights assumes that treatment is assigned at a single point in time by a single government regulator. This assumption fits well the empirical context of the intervention we evaluate—the 1987 Cabinet resolution in Thailand—but we acknowledge that interventions in other contexts can be implemented at different times and by different regulators. Future research could work on relaxing this limitation by extending the method to multiple treatments, as has been done for example with difference-in-differences models. We note that mangrove management policy in Thailand continued to evolve after 2000 (Beresnev et al. 2016) and that our findings for protection during 1987-2000 should not be assumed to apply to the subsequent period. Future research could also work on relaxing a third limitation: the method models a situation where the utility impacts of treatment are spatially independent. The benefits of forest protection are instead often spatially interdependent, with protection of site *i* supplying greater benefits if site *j* is also protected. Examples include watershed conservation and wildlife habitat. Non-environmental treatments might similarly have spatially interdependent impacts.

The Thailand application reveals a fourth limitation: distinguishing estimates of the utility-weighted ATT and the ATT for avoided deforestation from each other is less feasible in smaller samples. Both estimates were highly significant individually, thus indicating that protection reduced deforestation and had a positive economic impact (as valued by the government). Moreover, the utility-weighted ATT was substantially smaller than the ATT for avoided deforestation. Yet, the two estimates were not significantly different. To estimate the difference between them more precisely, we would have needed a larger number of treated observations to overcome the large standard errors that can result from the variation in the utility weights.

We close by noting a difference in how a government might respond to a low estimate of the utility-weighted ATT compared to a low estimate of the physical ATT, when the government’s objective is to maximize social welfare. The response to a low estimate of the physical ATT could include shifting program targets to alternative sites (or recipients), in order to increase a program’s impact as measured in physical terms. The theoretical framework for the utility-weighted ATT does not imply a similar option to retarget a program, because it assumes that the government made the socially optimal choice of program targeting when it selected sites (or recipients) for treatment. If a program’s impact as measured by the utility-weighted ATT is low, then increasing its impact is purely a matter of strengthening enforcement, not retargeting. Viewed from another angle, this difference implies that retargeting a program in response to a low estimate of the physical ATT might not be welfare-improving, particularly when the estimates of the physical ATT and the utility-weighted ATT are very different.

**Table 1:** $\tau _{ATT^Q}$ and $\tau _{ATT^W}$ in a simple numerical example

Site	Effective protection		
	Case 1	Case 2	Case 3
A (Area = 1, Weight = 1)	Yes	No	No
B (Area = 1, Weight = 2)	No	Yes	No
C (Area = 1, Weight = 3)	No	No	Yes
$\tau _{ATT^Q}$	1/3	1/3	1/3
$\tau _{ATT^W}$	1/6	1/3	1/2

**Table 2:** Protection zone criteria–1987 cabinet resolution

Criterion	Content
1.1	Areas designated as sanctuaries for aquatic plants and animals
1.2	Spawning grounds for aquatic animals and regeneration grounds for aquatic plants
1.3	Areas vulnerable to erosion such as beaches, sand dunes, and mudflats
1.4	Areas of historical and archaeological importance
1.5	Areas with locally unique features
1.6	National parks, forest parks, tourism areas, wildlife sanctuaries, and non-hunting areas
1.7	Areas that should be preserved as wind breaks
1.8	Areas that should be preserved for research purposes
1.9	Areas that should be preserved for environmental ecosystem protection
1.10	Areas that are within 20 meters of natural water channels such as rivers and natural canals and are not more than 75 meters from the coast line

**Table 3:** Definitions and summary statistics of the covariates

Covariate	Units	Treated points (N = 671)		Untreated points (N = 7119)	
		Mean	Std. Dev.	Mean	Std. Dev.
Distance to river	Meters	795	1,051	576	747
Distance to coast	Meters	1,725	2,340	2,634	2,655
Wind speed: high	Binary	0.246	0.431	0.241	0.428
Wind speed: low	Binary	0.250	0.434	0.343	0.475
Climate: non-monsoon	Binary	0.037	0.190	0.216	0.412
Animal species	Principal component	−1.18	2.23	−0.25	1.69
Gulf of Thailand	Binary	0.188	0.391	0.526	0.499
Distance to province capital	Meters	32,498	18,450	29,905	19,312
Distance to district seat	Meters	14,380	7,770	11,257	6799
Distance to road	Meters	5,489	3,817	4,337	3,167
Distance to edge: near	Binary	0.250	0.434	0.526	0.499
Distance to edge: far	Binary	0.249	0.433	0.103	0.304
Population density	People per km^2^	68.4	46.8	126.8	103.8
Mangrove area share	Share	0.238	0.149	0.220	0.153

**Table 4:** Covariate balance with and without matching (match number $=$ 2)

Variable	Standardized difference		Variance ratio	
	Raw	Matched	Raw	Matched
Distance to river	0.186	− 0.041	1.938	1.046
Distance to coast	− 0.341	− 0.075	0.793	0.905
Wind speed: high	0.056	0.036	1.055	1.033
Wind speed: low	− 0.204	− 0.056	0.890	0.959
Climate: non-monsoon	− 0.553	− 0.016	0.220	0.929
Animal species	− 0.422	− 0.020	1.699	0.949
Gulf of Thailand	− 0.737	0.040	0.631	1.066
Distance to province capital	0.146	0.039	0.941	0.985
Distance to district seat	0.374	0.063	1.223	1.000
Distance to road	0.292	− 0.010	1.396	1.042
Distance to edge: near	− 0.599	− 0.020	0.896	0.984
Distance to edge: far	0.413	0.062	1.963	1.069
Population density	− 0.760	0.002	0.372	0.987
Mangrove area share	0.164	− 0.035	0.937	0.943
*Predicted propensity score*	1.025	0.007	2.929	1.027

**Table 5:** Propensity score matching estimates with controls for spillovers

Exclusion criteria	$\tau _{ATT^Q}$	N1	N0
Baseline: No Exclusion	0.173***	646	7,101
	(0.024)		
Exclusion distance: 1 km	0.199***	618	6,023
	(0.027)		
Exclusion distance: 2 km	0.192***	625	4,972
	(0.028)		
Exclusion distance: 3 km	0.189***	607	4,098
	(0.031)		
Exclusion distance: 4 km	0.197***	553	3,534
	(0.029)		
Exclusion distance: 5 km	0.192***	437	2,950
	(0.034)		

Standard errors in parentheses

*p < 0.1, **p < 0.05, ***p < 0.01

N1 and N0 indicate the numbers of observations in the treated group and the untreated group, respectively

**Table 6:** Results of the logistic regression on treatment decision

Variable	Marginal effect	Std. err.
Distance to river	1.07E−05	1.44E−05
Distance to coast	2.38E−06	5.74E−06
Wind speed: high^a^	− 0.0198	0.0259
Wind speed: low^a^	− 0.0449*	0.0259
Climate: non-monsoon^a^	− 0.0626	0.0428
Animal species	− 0.0150***	0.00491
Gulf of Thailand^a^	− 0.0709*	0.0420
Distance to province capital	− 8.91E−07	7.71E−07
Distance to district seat	− 7.97E−07	1.63E−06
Distance to road	2.42E−07	2.57E−06
Distance to edge: near^a^	− 0.0599***	0.00942
Distance to edge: far^a^	0.0361***	0.0125
Population density	− 0.000668**	0.000294
Mangrove area share	− 0.00269	0.0828
Observations	7790	
Pseudo R-square	0.181	

*p < 0.1, **p < 0.05, ***p < 0.01

^a^ Marginal effect is for the discrete change of dummy variable from 0 to 1

## Appendix 1: Derivation of the Weight $W_i$

For convenience, we simplify notation by denoting $U^S_i$ by $U_i$ and $X_i$ by *x*. The cumulative distribution function (cdf) of *U*, evaluated at a specific value *u*, is given by the standard logistic distribution,

24 $$\begin{aligned} F_U(u) = \frac{e^u}{e^u + 1} \end{aligned}$$

with corresponding probability density function (pdf)

25 $$\begin{aligned} f_U(u) = \frac{e^{-u}}{(1+e^{-u})^2} \end{aligned}$$

Note that $\mathbb {E}[\beta x + U_i|\beta x + U_i > 0]$ is the expectation of the distribution $\beta x + U_i$ truncated at $\beta x + U_i > 0$. Since $U_i$ has an independent and identical distribution, we can further simplify notation by dropping the index *i* and defining $Z(x) = \beta x + U$. The distribution of *Z*(*x*) is the distribution of *U* shifted to the left by $\beta x$. Since *U* has a standard logistic distribution, we can write the pdf of *Z*(*x*) as

26 $$\begin{aligned} f_{Z(x)}(z) = \frac{e^{-(z-\beta x)}}{(1+e^{-(z-\beta x)})^2} \end{aligned}$$

The corresponding cdf for *Z*(*x*) is

27 $$\begin{aligned} F_{Z(x)}(z) = \frac{e^{z - \beta x}}{e^{z - \beta x} + 1} \end{aligned}$$

The probability that $Z(x)>0$ is therefore

28 $$\begin{aligned} \mathbb {P}(Z(x)>0) = 1 - F_{Z(x)}(0) = 1 - \frac{e^{-\beta x}}{e^{-\beta x} + 1} = \frac{1}{e^{-\beta x} + 1} \end{aligned}$$

Now, we can derive the expectation of the right tail of *Z*(*x*), truncated at $Z(x)>0$:

29 $$\begin{aligned} \begin{aligned} \mathbb {E} [Z(x)|Z(x)>0]&= \frac{\int _{0}^{\infty }z f_{Z(x)}(z) dz}{\mathbb {P}(Z(x)>0)}\\&= \frac{\int _{0}^{\infty }z \frac{e^{-(z-\beta x)}}{(1+e^{-(z-\beta x)})^2} dz}{\frac{1}{e^{-\beta x} + 1}}\\&= \frac{e^{\beta x}}{1 + e^{\beta x}} \int _{0}^{\infty } \frac{e^{\beta x} z e^{-z}}{(1 + e^{\beta x} e^{-z})^2} dz \end{aligned} \end{aligned}$$

Denoting $A = e^{\beta x}$ and $v = \frac{1}{1+Ae^{-z}}$, we have

30 $$\begin{aligned} dv&= \frac{Ae^{-z}}{(1+Ae^{-z})^2} dz \end{aligned}$$

31 $$\begin{aligned} z&= \ln A + \ln v - \ln (1-v) \end{aligned}$$

and therefore

32 $$\begin{aligned} \begin{aligned} \mathbb {E} [Z(x)|Z(x)>0]&= \frac{A}{1+A} \int _{1/(1+A)}^{1} [\ln A + \ln v - \ln (1-v)] dv\\&= \frac{A}{1+A} \Big [ \int _{1/(1+A)}^{1} \ln A dv + \int _{1/(1+A)}^{1} \ln v dv - \int _{1/(1+A)}^{1} \ln (1-v) dv \Big ]\\&= \frac{A}{1+A} \ln (1+A)\\&= \frac{e^{\beta x}}{1+e^{\beta x}} \ln (1+e^{\beta x}) \end{aligned} \end{aligned}$$

which corresponds to Eq. (19) in the text.

## Appendix 2: Analytic Framework for $\hat{\tau }_{ATT^W}$ with continuous *Q*

Here we consider the case in which the observed physical outcome *Q* is continuous, such as hectares of forest. This outcome has an unobservable, heterogeneous social welfare value, $U_i(Q)$. Instead of estimating the ATT in physical terms,

33 $$\begin{aligned} \hat{\tau }_{ATT^Q} = \sum _i \frac{D_i}{\sum _i D_i} (Q_{i, D_i=1} - Q_{i, D_i=0}) \end{aligned}$$

we are interested in estimating the ATT in welfare terms,

34 $$\begin{aligned} \hat{\tau }_{ATT^U} = \sum _i \frac{D_i}{\sum _i D_i} (U_i(Q_{i, D_i=1}) - U_i(Q_{i, D_i=0})) \end{aligned}$$

We proceed by assuming $U_i(Q)$ has the following structure:

35 $$\begin{aligned} U_i(Q_{i,D_i=1})&= \omega _{i1} Q \end{aligned}$$

36 $$\begin{aligned} U_i(Q_{i,D_i=0})&= \omega _{i0} Q \end{aligned}$$

A policy maker who acts on behalf of society does not observe $\omega$ but has an implicit or explicit model for predicting it by using a set of covariates *Z*,

37 $$\begin{aligned} \omega _{i1}&= f_1(Z_i) + \varepsilon _{i1} \end{aligned}$$

38 $$\begin{aligned} \omega _{i0}&= f_2(Z_i) + \varepsilon _{i0} \end{aligned}$$

The first moment of the difference of these two expressions, $\omega _i = \mathbb {E}[\omega _{i1}] - \mathbb {E}[\omega _{i0}] = f_1(Z_i) - f_2(Z_i)$, is ex ante expected welfare (as viewed by the policy maker) from treating site *i* when the physical outcome increases by one unit. Assuming treatment can only change *Q* in one direction (e.g., it can only increase, not decrease, forest area), the policy maker treats *i* ($D_i = 1$) if and only if $\omega _i>0$. We can further decompose $\omega _i$ into two components, $\omega _i = \theta X_i + \epsilon _i(Z_i|X_i)$, where *X* is a subset of *Z* that is correlated with the physical outcome *Q* and is observable by econometricians. This decomposition implies that the CIA is satisfied: $D|X \perp (Q_{i, D_i=1}, Q_{i, D_i=0})$. We can use propensity score matching to control for *X*, and we can estimate the weight, $W_i = \mathbb {E} [\omega _i | \omega _i>0]$, using observable data on *D* and *X*. These steps allow us to rewrite

39 $$\begin{aligned} \hat{\tau }_{ATT^W} = \sum _i \frac{D_i W_i}{\sum _i D_i W_i} (Q_i - Q_j) \end{aligned}$$

which corresponds to the expression derived in the text for a binary *Q*.

## Appendix 3: The Standard Error of $\hat{\tau }_{ATT^W}$

Estimating the standard error of matching estimators is challenging. It requires establishing an asymptotic approximation to the distribution of matching estimators, which is difficult because the estimators are non-smooth functions of the distribution of matching variables. Furthermore, Abadie and Imbens (2008) have shown that, for a fixed number of matches, bootstrapping is not valid for matching estimators. We therefore follow the alternative method for estimating standard errors of treatment effect estimators that Abadie and Imbens (2006) developed. First, we rewrite Eq. (22) as

40 $$\begin{aligned} \hat{\tau }_{ATT^W} = \sum _{i=1}^n \hat{w}_i \hat{\tau }_i \end{aligned}$$

where $\hat{w}_i = D_i \hat{W}_i / \sum _i D_i \hat{W}_i$ and $\hat{\tau }_i = Q_i - Q_j$. We treat $\hat{w}_i$ as given, i.e., $\hat{w}_i = w_i$, so the variance of $\hat{\tau }_{ATT^W}$, conditional on the covariates and the treatment indicators, can be expressed as

41 $$\begin{aligned} V[\hat{\tau }_{ATT^W}|X, D] = V[\sum _{i=1}^n \hat{w}_i \hat{\tau }_i|X, D] = \sum _{i=1}^n w^2_i V[\hat{\tau }_i|X, D] = \sum _{i=1}^n w^2_i \big ( \sigma ^2_1(X_i) + \sigma ^2_0(X_j) \big ) \end{aligned}$$

This procedure is similar to the way that Abadie and Imbens (2006) establish the variance of the conventional ATT matching estimator. We need to estimate $\sigma ^2_1(X_i)$ for all *i* in the treatment group and $\sigma ^2_0(X_j)$ for all *j* that are used as the matched controls. Fortunately, these estimates do not need to be consistent. Abadie and Imbens (2006) suggest estimating them using matching within the treatment group and the control group, respectively, and we implement their method.

In a more recent study, Abadie and Imbens (2016) show that the variance of matching estimators must be adjusted for the fact that propensity scores are estimated prior to matching. They derive the adjustment term, and they develop a procedure to estimate it. The adjustment term, denoted as $\sigma ^2_{adj}$, is based entirely on the first-stage regression that estimated the propensity scores, which is identical to our first stage in estimating $\hat{\tau }_{ATT^W}$. Therefore, we can directly add the adjustment term to the estimation of the variance of $\hat{\tau }_{ATT^W}$ given by Eq. (41):

42 $$\begin{aligned} \hat{V}[\hat{\tau }_{ATT^W}|X, D] = \sum _{i=1}^n \hat{w}^2_i \big ( \hat{\sigma }^2_1(X_i) + \hat{\sigma }^2_0(X_j) \big ) + \hat{\sigma }^2_{adj} \end{aligned}$$

When $\hat{\sigma }^2_1(X_i)$ and $\hat{\sigma }^2_0(X_j)$ are homogeneous across *i* and *j*, the variance of $\hat{\tau }_{ATT^W}$ is always larger than the variance of the corresponding $\hat{\tau }_{ATT^Q}$ due to the presence of unequal weights. In that particular case, the economically more meaningful measure of a program’s welfare impact that $\hat{\tau }_{ATT^W}$ provides compared to $\hat{\tau }_{ATT^Q}$ comes at the cost of reduced precision. In the general case, however, the variance of the individual treatment effect can be heterogeneous. Consequently, the variance of $\hat{\tau }_{ATT^W}$ can be larger, smaller, or equal to the variance of $\hat{\tau }_{ATT^Q}$, depending on how weights are assigned.

The variance formula for $\hat{\tau }_{ATT^W}$ developed above has a limitation, as it treats $\hat{w}_i$ as given weights. Analyzing the distribution of a weighted average of random variables when the weights themselves are random is a challenging issue that remains on the frontier of statistical research (Tang and Yuan 2014; Roozegar and Soltani 2015). We do not attempt to solve that issue in this paper.

## Appendix 4: Data Sources and Variable Construction

### Mangrove Cover

We acquired GIS data on 2000 mangrove cover in Thailand from the Global Mangrove Forest Distribution, v1 (2000) dataset (Giri et al. 2005, 2011). This dataset is in raster format, with a 30-meter pixel size. It codes mangrove cover as a binary variable with 1 indicating mangrove presence and 0 indicating absence. We created corresponding 30-meter resolution data on 1987 mangrove cover using the same source of satellite images (Landsat) and the same techniques used by Giri et al. (2011). We obtained Top-Of-Atmosphere (TOA) Landsat images for 1987 from the Google Earth Engine archive. If 1987 images were not available or were obscured by clouds, then we substituted images from 1986 or 1988. Such substitution represented less than 5% of the data used. We analyzed the images in the cloud computing environment of Google Earth Engine. We selected approximately 10,200 training points for three surface-cover classes: mangrove, non-mangrove, and water. We based selection of training points on several factors, including a shapefile for the boundaries of the zoning system (see below), very high-resolution satellite data (WorldView, GeoEye), and the spectral signature of the Landsat data (bands 1–5 and 7). We classified the Landsat data using a supervised classification approach with a random forest classifier. We performed the classification separately for 19 regions of interest (ROIs) that spanned the entire coastal region of Thailand. We classified the ROIs one at a time, with training images selected from within the same ROIs to improve classification accuracy. We edited the data in ERDAS IMAGINE, removing obvious errors such as false classification of mangroves in hilly areas. We converted the edited data so that it included the mangrove class only, and as a final step we mosaicked all the ROIs together to form a single, 1-bit dataset for the entire country. This dataset is available from the corresponding author.

### Treatment Status

The Thailand Department of Marine and Coastal Resources (DMCR) provided a shapefile containing the boundaries of the Protection Zone, Economic Zone A, and Economic Zone B from the RFD map associated with the 1987 Cabinet resolution. We used GIS to overlay the boundaries onto our sample of points. We used a binary variable to code the treatment status, with observations in the Protection Zone coded as the treated group ($D=1$) and observations not in any of the zones coded as the untreated group ($D=0$).

### Pre-treatment Protected Areas

We generated a binary variable that identified mangroves that were already within national parks and other PAs when the zoning system was introduced. The World Database on Protected Areas (WDPA) has information on PAs in Thailand, including their establishment dates and GIS polygons of their boundaries (UNEP-WCMC and IUCN 2019). We selected coastal PAs in the WDPA that were established no later than 1987, and we created a binary variable that equaled 1 if a point in our sample was within any of them and equaled 0 otherwise. We used features on the Google Earth base map to manually correct the boundaries of the PAs, as most of the polygons were displaced by $\sim$0.45 km in a southeasterly direction, and some were displaced by up to $\sim$1 km in other directions. All of the coastal PAs for Thailand in the WDPA were national parks with the exception of three non-hunting areas (Thale Noi, Thale Sap, Mu Ko Libong). The WDPA did not include polygons for those three areas, but we obtained polygons for them from the Thailand Department of National Parks, Wildlife, and Plant Conservation. The Department also provided polygons that enabled us to correct additional boundary errors in the WDPA polygons for several Marine National Parks (Had Chao Mai, Had Noparatthara—Mu Ko Phi Phi, Khao Lam Pi—Hat Thai Muang, Khar Sam Roi Yot).

### Covariates

We used tools in ArcMap to generate the covariates in the selection model. Throughout the analysis, we used GCS_WGS_1984 for geographical coordinates and Azimuthal Equidistant as the projection. We used the latter projection to minimize distance measurement errors caused by map distortions. We set the latitude-longitude of the center of the Azimuthal Equidistant projection at $10^{\circ } N$, $100^{\circ } E$, which is approximately at the center of our study area.

*Distance to river*. We measured distance to the nearest river using a stream map of Thailand available on Marc Souris’s research website.^11^ We visually checked the map by overlaying it on the Google Earth base map, which revealed that it omitted many tidal streams near river mouths. Before taking the distance measurements, we manually edited the map to add tidal streams shown on the Google Earth base map.

*Distance to coast*. We evaluated two sources of data on Thailand’s coastline: the shoreline map in the Global Self-consistant Hierarchical, High-resolution Geography (GSHHG) database (Wessel and Smith 1996);^12^ and a digital map of administrative boundaries in Thailand that we obtained from the DMCR. We selected the latter because it matched much more closely with the mangrove zone boundary map. We visually checked its accuracy by overlaying it on the Google Earth base map. This comparison prompted us to manually edit one part of the administrative boundaries map (i.e., Thailand’s international boundary with Myanmar along the Kra Buri River). We then used ArcMap to calculate the distance between each point in the sample and the nearest coastline. Because some mangroves can be submerged even during low tide, some points were classified as being beyond the coastline. We recoded distances for these points as 0 (i.e., directly on the coastline).

*Wind speed*. We drew data on wind speed from the Global Wind Atlas 2.0.^13^ We extracted the 50-meter aboveground wind speed as a raster file from this database and overlaid it on our sample of points. To improve balance, we discretized the variable by converting it to a pair of dummy variables, one for points with wind speeds above the 75th percentile of the treated group (*Wind speed: high*) and the other for points with wind speeds below the 25th percentile (*Wind speed: low*). We also considered including a variable on cyclone frequency, but global data on cyclone frequency show little variation in Thailand (CHRR 2005, Dilley et al. 2005).

*Climatic zones*. We generated categorical dummy variables for climatic zones. We manually coded a map of the World K$\mathrm {\ddot{o}}$ppen Climate Classification at the subdistrict (*tambon*) level.^14^ Our study area includes only three climatic zones: equatorial, monsoon, and tropical savanna. We combined the dummies for the equatorial and tropical savanna zones into a single variable, as less than 4% of the treated points were in these two zones. The reference (excluded) zone in the selection model was thus the monsoon zone.

*Mangrove-resident animal species*. We considered several data sources on the distribution of mangrove-resident animal species in Thailand. We focused on amphibians, birds, and mammals, whose geographical distributions are better known than for other taxonomic groups. One source was the International Union for Conservation of Nature (IUCN), whose Red List website provides comprehensive data on threatened species throughout the world. An obstacle to using these data was that we wanted data on the geographical distributions of species that were threatened in 1987 (or a year close to it), but IUCN reports current threat status. We therefore instead used data on the total number of species, not the number of threatened species. We acquired raster data on geographical distributions of amphibian species from IUCN and CIESEN (International Union for Conservation of Nature 2015a) and mammal species from IUCN and CIESEN (International Union for Conservation of Nature 2015b). We acquired corresponding data on bird species from BirdLife International and Handbook of the Birds of the World (BirdLife International and Handbook of the Birds of the World. 2018). We determined the number of species in each of the three taxonomic groups at each point in our sample by intersecting the species distributions with the coordinates of the points. We used the total historical range for each species, which refers to the area where the species is known or thought very likely to occur presently or historically. The covariate in the selection model is the first principal component of the variables for the three taxonomic groups. The first principal component contains 87% of the variation in the three variables.

*Coasts*. Mangroves occur on both of Thailand’s coasts (Andaman Sea, Gulf of Thailand). Using the administrative boundaries map from the DMCR, we determined the province where each point in our sample was located and then classified the provinces by coast. No province is on both coasts. We included the dummy for the Gulf of Thailand coast in the selection model.

*Distances to cities and roads*. We used ArcMap to generate several other distance variables that are commonly included as covariates in the matching literature on forest conservation programs. These variables are proxies for proximity to centers of economic or political influence, which might affect treatment status, outcomes, or both. The first of these variables was *distance to the provincial capital*. We compiled from Wikipedia and verified (against Thai sources) a list of the capitals of coastal provinces in Thailand and their geographical coordinates. Having determined the province where each point in our sample was located, we calculated the distance from each point to the corresponding provincial capital. The second variable was *distance to the district seat*. Districts (*amphoe*) are one level below provinces in Thailand’s administrative structure. We constructed this variable in the same way as distance to the provincial capital. The last distance variable was *distance to the nearest road*. The Digital Chart of the World includes a global road map for 1992.^15^ We used this map to measure the distance between each point in our sample and the nearest road in Thailand as of that year. The data on distances to nearest urban area and nearest road are post-1987, which could cause them to be endogenous. The risk of endogeneity is small, however, because these variables are slow-moving and the number of years post-1987 is small.

*Distance to mangrove edge*. We used GIS tools and the 1987 mangrove layer to compute the distance between each point in our sample and the nearest terrestrial non-mangrove point. To improve balance, we discretized the variable by converting it to a pair of dummy variables, one for points with distances above the 75th percentile of the treated group (*Distance to edge: far*) and the other for points with distances below the 25th percentile (*Distance to edge: near*).

*Mangrove area share*. We used our 1987 mangrove cover dataset, the administrative boundaries map from the DMCR, and GIS tools to determine the total area of mangrove in the subdistrict where each point was located. We similarly determined the total area of each subdistrict, and we generated the mangrove area share variable as the ratio of the two areas.

*Population density*. Thailand’s National Statistical Office (NSO) conducts Population and Housing Censuses every decade, with the 1980 and 1990 censuses straddling the 1987 Cabinet Resolution. Published census documents for these years report data only at a highly aggregated level (i.e., province). The NSO informed us that it could not provide data at the disaggregated level we preferred (i.e., subdistrict) for either year. It advised us to request data instead from the Department of Local Administration, which has generated annual population estimates for subdistrict since 1993. The Department provided us the 1993 data, which we used to generate a population density variable for the subdistrict where each point was located. Given that 1993 is post-treatment, this variable could be endogenous. The risk of endogeneity is small, however, because the Protection Zone accounted for only 1.6% of the total area of the subdistricts where this zone was located. This tiny percentage makes it highly unlikely that the 1987 classification of mangroves into the Protection Zone significantly influenced subsequent human population changes. To improve balance, we winsorized the bottom and top tenths of the distribution of the population density variable (Barnett and Lewis 1994).

## Appendix 5: Covariate Balance when Match Number is 1

Here we present the covariate balance table with and without matching when we match each treated point with a single untreated point.

**Table:**

Variable	Standardized difference		Variance ratio	
	Raw	Matched	Raw	Matched
Distance to river	0.206	− 0.039	1.981	1.005
Distance to coast	− 0.347	− 0.030	0.788	0.970
Wind speed: high	0.066	0.148	1.064	1.165
Wind speed: low	− 0.210	− 0.049	0.885	0.963
Climate: non-monsoon	− 0.554	− 0.008	0.217	0.963
Animal species	− 0.436	0.021	1.710	0.920
Gulf of Thailand	− 0.742	0.004	0.626	1.006
Distance to province capital	0.139	0.004	0.934	1.020
Distance to district seat	0.390	0.010	1.236	0.922
Distance to road	0.291	− 0.070	1.390	1.063
Distance to edge: near	− 0.606	− 0.059	0.890	0.956
Distance to edge: far	0.425	0.045	1.981	1.047
Population density	− 0.763	0.018	0.369	1.067
Mangrove area share	0.153	− 0.048	0.939	0.928
*Predicted propensity score*	1.029	0.004	3.035	1.015

## Footnotes

1 An early valuation study on Thailand’s mangroves by Sathirathai (1998) was sponsored by the Economy and Environment Program for Southeast Asia (EEPSEA), which is affiliated with the Mäler Scholars Program that the Beijer Institute established in honor of Karl-Göran Mäler.

2 Sites that will be deforested only if they are treated do not exist given the assumption that regulation creates an additional private deforestation cost, $C>0$.

3 Chatree Maknual (Director, Mangrove Research and Development Division) and Traithip Phromthong (Director, Mangrove Conservation Division), interviewed at DMCR headquarters in Bangkok on June 18, 2019.

4 We also dropped points from a small part of Krabi Province that lacked mangrove data for 2000; those points accounted for less than 2% of the sample. They lacked 2000 data due to a technical oversight during image processing when Giri et al. (2005, 2011) created the 2000 map, so there is no reason to expect that selection into treatment or mangrove cover dynamics during 1987–2000 in this small region differed systematically from other parts of the country.

5 The text for this criterion in the 1987 Cabinet resolution contains an apparent error, as it states that mangroves should be “at least” X meters from these water features instead of “within” X meters of them. “Within,” not “at least,” was the term used by the DMCR officers when they explained the Protection Zone criteria.

6 The 2004 Indian Ocean tsunami elevated recognition of the coastal protection service of mangroves in Thailand (Barbier 2008).

7 Unlike Miteva et al. (2015), we did not include variables on household income or occupation. Household data for the 1980s are scarce in Thailand, which has not been the site of a World Bank Living Standards Measurement Study (https://microdata.worldbank.org/index.php/catalog/lsms). The Townsend Thai Project (http://townsend-thai.mit.edu/) did not launch its household surveys until 1997, and they include only two of the 24 Thai provinces with mangroves. Data from the annual Household Socio-Economic Survey conducted by Thailand’s National Statistical Office are available online only since 2001 and only for five aggregate regions (http://www.nso.go.th/sites/2014en/Pages/survey/Social/Household/The-2017-Household-Socio-Economic-Survey.aspx), several of which mix mangrove and non-mangrove provinces.

8 Miteva et al. (2015) included these variables in their study on mangrove protection in Indonesia because their units of observation were entire villages, which included interior regions and not only mangroves.

9 Although this statement refers to the impact of solely the Protection Zone, cumulative deforestation rates during 1987-2000 suggest that the overall zoning system might have had a similar impact: deforestation rates were virtually identical in the two zones that prohibited deforestation, the Protection Zone (17%) and Economic Zone A (18%); while the rate in the zone that allowed deforestation, Economic Zone B (48%), was identical to the rate in the unzoned area (48%), where the zoning system’s restrictions did not apply.

10 The ATT for avoided deforestation is slightly negative for two of the quantiles, which is likely due to imperfect precision of the estimated treatment effects and not the Protection Zone causing deforestation to occur.
